# The use of the Medication Event Monitoring System (MEMS) for assessing medication adherence for chronic conditions: use and results from a 12 month trial of patients in remission with ulcerative colitis (UC)

**DOI:** 10.1186/1745-6215-12-S1-A130

**Published:** 2011-12-13

**Authors:** David Gillespie, Kerenza Hood, Andrew Williams, Rachel Stenson, Christopher Probert, Antony Hawthorne

**Affiliations:** 1 South East Wales Trials Unit, School of Medicine, Cardiff University, UK; 2 Gastroenterology, University Hospital of Wales, UK; 3 School of Clinical Sciences, University of Bristol, UK

## Background

The Colitis Once Daily Asacol® (CODA) study assessed the efficacy and safety of once daily (OD) dosing with Mesalazine (Asacol®, 2.4g as 3 x 800mg tablets) versus one 800mg tablet three times daily (TDS) over a 12 month period for patients in remission with ulcerative colitis (UC). Emerging evidence suggests OD Mesalazine is as effective as divided doses [1]. While this has been attributed to better adherence, detailed measures of adherence have been lacking in previous studies. A sub study was run alongside in order to provide a more intense monitoring of adherence using the Medication Event Monitoring System (MEMS).

## Objectives

To (i.) describe the use of the MEMS for a detailed assessment of medication adherence and (ii.) present results from the CODA sub study, comparing adherence data collected using the MEMS with the methods used in the main trial.

## Methods

The main CODA trial collected tablet counts at patient visits and asked questions on perceived adherence. The CODA sub study used the MEMS cap data, which electronically recorded the time and date of each cap opening. It was assumed that each cap opening represented a patient taking the correct medication from the bottle [2].

## Results

A total of 58 patients had usable adherence data (49 with complete data (12 months or until relapse), 9 with partial data, 3 patients were withdrawn). The frequency of cap openings split by trial arm will be presented. The percentage of days adherent was significantly different between the two trial arms, with OD patients considerably more adherent than TDS patients. The impact of controlling for adherence on relapse rates will be presented. A comparison will be made between the MEMS adherence data and the adherence data obtained in the main trial. More detailed analysis of patient adherence; including (i.) weekday versus weekend adherence; (ii.) adherence around visit dates versus regular adherence and (iii.) patterns of adherence over time will be considered.

## Conclusions

Collecting adherence data electronically using products such as the MEMS provides an adequate representation of the complexities of patient adherence that may not be possible to obtain through other means (e.g. tablet counts and patient perception).
